# Functional Profiling of *Enterococcus* and *Pediococcus* Strains: An In Vitro Study on Probiotic and Postbiotic Properties

**DOI:** 10.3390/microorganisms13061348

**Published:** 2025-06-10

**Authors:** Mircea-Cosmin Pristavu, Filofteia Camelia Diguță, Alexandru Constantin Aldea, Florentina Badea, Mihaela Dragoi Cudalbeanu, Alina Ortan, Florentina Matei

**Affiliations:** 1 Faculty of Biotechnologies, University of Agronomic Sciences and Veterinary Medicine of Bucharest, 59 Marasti Blvd., 011464 Bucharest, Romania; cosmin_mircea96@yahoo.com (M.-C.P.); alexandru.aldea@bth.usamv.ro (A.C.A.); badea_florentina2003@yahoo.com (F.B.); florentina.matei@unitbv.ro (F.M.); 2 Faculty of Land Reclamation and Environmental Engineering, University of Agronomic Sciences and Veterinary Medicine of Bucharest, 59 Mărăsti Blvd., 011464 Bucharest, Romania; mcudalbeanu@gmail.com (M.D.C.); alina.ortan@fifim.ro (A.O.); 3 Faculty of Food Industry and Tourism, Transilvania University of Brașov, 148 Castelului Street, 500014 Brașov, Romania

**Keywords:** *Pediococcus acidilactici*, *Pediococcus pentosaceus*, *Enterococcus faecium*, probiotic potential, postbiotic potential

## Abstract

The rising threat of antimicrobial resistance (AMR) has driven the search for safe and effective alternatives to conventional antibiotics. This study investigated the probiotic potential and postbiotic properties of *Enterococcus faecium* (one strain), *Pediococcus acidilactici* (five strains), and *Pediococcus pentosaceus* (five strains), identified by 16S rRNA sequencing. Among the strains, ***Pediococcus pentosaceus* MI124 and *Pediococcus acidilactici* MI129** demonstrated robust survival under simulated gastrointestinal conditions. Cell surface analyses revealed strong auto-aggregation and hydrophobicity in selected strains, notably *P. pentosaceus* MI124 and *P. acidilactici* MI127. Enzymatic profiling revealed potential complex metabolic capabilities across different strains. Safety assessments confirmed the absence of hemolytic and gelatinase activities across all strains. Antibiotic susceptibility testing showed resistance to certain β-lactams, while susceptibility to chloramphenicol and tetracycline varied. All LAB strains demonstrated high freeze-drying survivability, exceeding 78.69%. The antibacterial activity of CFSs was confirmed against 14 Gram-positive and Gram-negative pathogens, with results supporting their potential as antimicrobial agents. The CFSs demonstrated a higher total phenolic content (TPC) and displayed significant antioxidant activity, while the total flavonoid content (TFC) remained consistent across most strains. An FTIR spectral analysis confirmed the presence of key functional groups associated with phenolics, organic acids, and peptides, indicating a complex biochemical profile. Probiotics and their postbiotic derivatives offer promising health benefits, including pathogen inhibition and immune modulation. These findings highlight several LAB strains with promising probiotic traits and postbiotic efficacy, supporting their potential use in functional foods and therapeutic applications.

## 1. Introduction

The swift rise and global spread of antimicrobial resistance (AMR) have severely burdened healthcare systems worldwide, with estimates suggesting that it could cause up to 10 million deaths per year by 2050 [1,2,3]. AMR plays a key role in the emergence of multidrug-resistant organisms, significantly reducing the effectiveness of standard antibiotic treatments [3]. Moreover, the indiscriminate use of broad-spectrum antibiotics can perturb the natural balance of the gut microbiota, potentially leading to various health issues [1,2,3].

As a result, efforts have intensified to explore safe and effective alternatives to traditional antibiotic therapies [4]. The FAO/WHO defines probiotics as “live microorganisms which, when taken in appropriate amounts, provide a health benefit to the host” [5,6]. They have attracted significant interest due to their ability to promote gut health, regulate immune function, combat pathogenic microbes, and generate beneficial bioactive compounds [4,7,8,9,10]. Among lactic acid bacteria (LAB), genera such as *Bifidobacterium* and *Lactobacillus* are well known for their probiotic roles [11,12,13,14,15,16,17]. Research also notes that *Saccharomyces* and non-*Saccharomyces* have been considered for probiotic use [18,19,20,21]. Despite the increasing recognition of the functional potential of *Enterococcus faecium, Pediococcus pentosaceus*, and *Pediococcus acidilactici,* a notable gap persists in the scientific literature regarding the characterization of the probiotic and postbiotic capabilities of these LAB species in food, pharmaceutical, and clinical contexts [22,23,24,25,26,27].

A microbial strain must meet several safety and functional criteria for probiotic use. Such criteria include, but are not limited to, the following: genetic stability, non-pathogenicity, resistance to gastrointestinal conditions (acid pH and gastric enzymes), resistance to processing-related stresses, and a rapid growth rate [28,29]. Probiotics generate beneficial effects by strengthening the epithelial barrier, facilitating attachment to intestinal epithelial cells while preventing pathogen adhesion, producing bioactive compounds that inhibit pathogen growth, and modulating immune system responses [29,30]. Fermented foods containing probiotics are recognized for their health benefits [14,15,16]. However, pathogenic infections, competition for nutrients from non-functional microflora, and environmental fluctuations can compromise the beneficial effects of these foods. Integrating postbiotics offers an innovative approach to improving food safety and optimizing health benefits [31,32,33,34,35,36,37,38,39]. In 2021, the ISAPP (International Scientific Association for Probiotics and Prebiotics) officially defined postbiotics as “preparations of inactivated microorganisms and/or their cellular components and metabolic by-products that confer health benefits to the host” [40,41]. According to FDA guidelines, postbiotics are categorized as Generally Recognized as Safe (GRAS), indicating that they are considered safe for consumption among all age groups, with minimal risk of side effects [33,36,41,42]. Postbiotics consist of a variety of bioactive substances, including enzymes, vitamins, bacteriocins, teichoic acids, muropeptides derived from peptidoglycan, exopolysaccharides, cell surface proteins, and short-chain fatty acids (SCFAs), all of which are released by beneficial microorganisms during growth and fermentation in complex environments, such as food or the gut [31,32,33,34,35,36,37,38]. These compounds’ antimicrobial, antioxidant, anti-inflammatory, and immunomodulatory properties are well established [33,37,41,42,43,44].

Despite these known benefits, there are still gaps in our understanding of how these substances can be effectively applied in food production. Recent research into preparing, separating, and using postbiotics from different probiotics is encouraging. However, further studies are needed to evaluate their efficacy in food safety [33,34,35,36]. This can be achieved by analyzing their chemical composition [38,45,46,47,48,49]. Unlike probiotics, postbiotics are stable under harsh environmental conditions and require minimal processing [33,40,41]. This improves food safety and broader consumer acceptance [38,45,46,47,48,49]. Therefore, postbiotics with clearly defined and thoroughly characterized effects have the potential to offer significant benefits to the food, pharmaceutical, and nutraceutical industries [31,32,33,34,35,36,37,38,39].

Although *Pediococcus* strains (*P. pentosaceus* and *P. acidilactici*) are included in the EFSA QPS list and show promise for producing bioactive compounds, further clarification is needed regarding their application as natural antimicrobials and bio-preservatives [50,51,52,53]. Similarly, *E. faecium*, while increasingly recognized for its antimicrobial and immunomodulatory properties, has yet to achieve QPS status due to insufficient strain-specific safety data and concerns about potential pathogenicity [54]. Addressing these gaps by thoroughly characterizing functional properties, safety profiles, and postbiotic activity is essential for utilizing these species as effective and safe alternatives to traditional probiotics, especially in combating antimicrobial resistance [51,52,53,54]. This research focused on conducting an extensive in vitro evaluation of *Enterococcus faecium, Pediococcus pentosaceus*, and *Pediococcus acidilactici* strains regarding their probiotic potential and postbiotic capabilities, aiming to identify promising candidates as alternative or complementary strategies to reduce reliance on conventional antibiotics.

## 2. Materials and Methods

### 2.1. Preparation of Bacterial Strains and Growth Conditions

Eleven LAB strains from the Microorganisms Collection of the Faculty of Biotechnologies (UASMV Bucharest), stored in a 40% glycerol solution at −20 °C, were utilized. The strains were initially transferred into a specific culture medium (De Man, Rogosa, and Sharpe (MRS) broth, sourced from Merck, Darmstadt, Germany) and incubated at 37 °C for 24 h. Subsequently, the strains were cultured on MRS agar medium (Merck, Darmstadt, Germany) and incubated for another 24 h at 37 °C.

The antimicrobial potential of cell-free supernatants (CFSs) obtained from the LAB strains was evaluated against a panel of six Gram-negative bacteria, namely, *Escherichia coli* ATCC 8739, *Proteus vulgaris* ATCC 13315, *Pseudomonas aeruginosa* ATCC 15442, *Salmonella enterica* serovar Typhimurium ATCC 14028, *Salmonella enterica* serovar Enteritidis ATCC 14028, and *Serratia marcescens* ATCC 14756, as well as eight Gram-positive strains, namely, *Bacillus cereus* ATCC 11778, *Kocuria kristinae* MI 20, *Listeria monocytogenes* ATCC 7644, *Staphylococcus aureus* ATCC 33592 (MRSA), *S. aureus* ATCC 6538, *S. epidermidis* ATCC 51625 (MRSE), *S. epidermidis* ATCC 12228, and *Rhodococcus equi* ATCC 6939. All reference strains were initially cultured on trypticase soy agar (TSA; Alliance Bio Expertise, Guipry Messac, France) at 37 °C for 24 h. One to two colonies were transferred into a 0.9% NaCl solution and standardized to 10^6^ CFU/mL according to McFarland turbidity guidelines.

### 2.2. Evaluation of Probiotic Properties of LAB Strains

#### 2.2.1. Identification of LAB Strains via 16S rDNA Sequencing

Freshly cultured LAB cells were collected by centrifugation at 5000× *g* for 10 min. Following the manufacturer’s protocol, genomic DNA was isolated using a Quick-DNA^TM^ Fungal/Bacterial Miniprep Kit (Zymo Research, Irvine, CA, USA). The 16S ribosomal DNA region was amplified for molecular identification using the universal primer set 27F and 1492R [55]. The PCR reaction mixture contained 10× DreamTaq Green Buffer (including 20 mM MgCl_2_), 0.5 µM of each primer, 0.2 mM dNTPs, and 0.25 U of DreamTaq DNA Polymerase (Thermo Fisher Scientific Baltics, UAB, Vilnius, Lithuania), with MilliQ water added to adjust the volume. The PCR protocol included an annealing step at 55.5 °C. Sequencing of the PCR products was performed by CEMIA (Cellular and Molecular Immunological Application, Larissa, Greece). The resulting partial sequences were matched with entries in the NCBI GenBank database (http://www.ncbi.nlm.nih.gov; accessed on 27 January 2025) to determine species-level identity based on sequence similarity.

#### 2.2.2. In Vitro Tolerance of LAB Strains to Simulated Gastric and Intestinal Conditions

The tolerance of LAB isolates to acidic conditions in the presence of pepsin was evaluated using the method described by Digută et al. [56]. Overnight LAB cultures were centrifuged at 2000× *g* for 5 min, and the pelleted cells were washed twice with a sterile 0.9% NaCl solution. The bacterial concentration was adjusted to approximately 1.0 × 10^8^ CFU·mL^−1^. The bacterial suspension was then resuspended in phosphate-buffered saline (PBS) (Central Drug House, Mumbai, India), with the pH adjusted to 2.5 using 1 M HCl solution and supplemented with 0.3% (*w*/*v*) pepsin (Himedia, Mumbai, India). The mixture was incubated at 37 °C for 3 h.

To assess bile salt tolerance, LAB cells harvested by centrifugation at 2000× *g* for 5 min were washed twice with a 0.9% NaCl solution, inoculated into MRS broth adjusted to pH 8.0 using 1 M NaOH, and supplemented with 0.3% (*w*/*v*) bile salts. The cultures were incubated at 37 °C for 4 h.

In both experiments, viable cell counts were assessed using the plate count method by collecting samples at the start (0 h) and after the respective incubation periods (3 h or 4 h). The survival rate under both stress conditions was calculated using the following formula:

$$SR\;\%=\frac{\log\;\mathrm{CFUe}\;}{\log\;\mathrm{CFUi}}\times 100$$

where CFUi and CFUe represent the colony-forming units at the start and end of incubation, respectively, under simulated gastrointestinal conditions.

#### 2.2.3. Phenol Tolerance

The phenol tolerance of the LAB isolates was assessed based on the method described by Kouadio et al. [13]. Fresh cultures, adjusted to approximately 10^7^ CFU·mL^−1^, were inoculated into MRS broth containing 0.4% phenol and incubated at 37 °C for 24 h. After incubation, the survival rate was calculated using the previously established formula.

#### 2.2.4. Cell Surface Characteristics

##### Cell Surface Hydrophobicity

Cell surface hydrophobicity, which reveals how LAB cells resist water and attach to non-polar materials, was measured using a modified version of the Rosenberg et al. [57] method. Overnight LAB cultures were collected by centrifugation at 4000× *g* for 10 min at 4 °C. The pellets were washed twice with PBS and adjusted to an optical density of 0.650 ± 0.20 at 600 nm (noted as H0). Hydrophobicity was tested by combining 2.4 mL of the suspension with 0.4 mL of hexane (VWR International, Rosny-sous-Bois, France) or xylene (Bernd Kraft GmbH, Wetzlar, Germany), followed by 2 min of intense vortexing. Following 30 min of incubation at room temperature to allow for phase separation, the aqueous phase was carefully collected, and its absorbance (noted as H1) was measured at 600 nm. The hydrophobicity percentage was then determined using the following formula:

$$H\;\%=\left(\frac{H0-\;H1}{H0}\right)\times 100$$

##### Auto-Aggregation Test

The ability of LAB isolates to auto-aggregate was assessed following the protocol described by Kouadio et al. [13]. Overnight LAB cultures were harvested by centrifugation at 4000× *g* for 10 min at 4 °C, washed twice with phosphate-buffered saline (PBS), and then resuspended in fresh PBS. These bacterial suspensions were incubated at 37 °C for 4 h. Absorbance was recorded at 600 nm initially (A0) and after incubation (At). The percentage of auto-aggregation was determined using the following formula:

$$AA\;\%=\left(1-\frac{\mathrm{At}}{A0}\right)\times 100$$

#### 2.2.5. Enzymatic Pattern of the LAB Strains

Enzymatic activity was assessed by introducing 10 μL of freshly cultured LAB into selective media and incubating at 37 °C for 72 h, based on the modified procedure outlined by Proca et al. [58]. Amylase activity was assessed on TSA supplemented with 0.4% soluble starch. Gram’s iodine visualized the zone of precipitation. Cellulase activity was tested on TSA containing 1% carboxymethylcellulose (CMC), followed by Congo red staining for 30 min and washing with 1 M NaCl. A clear zone around the colonies indicated cellulase production. Protease activity was tested on skim milk agar, with a clear zone indicating casein hydrolysis. Gelatinase activity was assessed on TSA with 3% gelatin, and the liquefaction of the medium after 30 min in an ice bath indicated positive gelatinase activity. Phytase activity was tested on a TSA medium containing sodium phytate, with phytase-producing strains identified by a clear zone around colonies. Catalase activity was assessed by placing a drop of 3% hydrogen peroxide (H_2_O_2_) on a microscope slide and adding a small sample of the fresh bacterial culture using a sterile inoculation loop. According to the manufacturer’s instructions, hydrolytic enzymes were also detected using an API ZYM kit (Biomèrieux, Montalieu-Vercieu, France). Enzyme activity was scored based on color intensity, with index values ranging from no activity (−) to weak activity (+), moderate activity (++), and strong activity (+++).

### 2.3. Safety Assessment

#### 2.3.1. Hemolysis Assay

The hemolytic properties of LAB isolates were examined using a method adapted from Coulibaly et al. [12]. An aliquot of 10 µL from each LAB culture was spotted onto blood agar plates (Oxoid, Basingstoke, Hampshire, UK) enriched with 5% (*w*/*v*) sheep blood. The plates were then incubated at 37 °C for 48 h. Hemolytic activity was classified as follows: γ-hemolysis was identified by the absence of any hemolysis or blood cell breakdown around the bacterial colonies, β-hemolysis was identified by the formation of a clear zone surrounding the bacterial colonies, and a greenish color in the surrounding area indicated α-hemolysis.

#### 2.3.2. Antibiotic Susceptibility Testing

The antibiotic susceptibility of LAB strains was evaluated using the disc diffusion method, following guidelines provided by the European Committee on Antimicrobial Susceptibility Testing (EUCAST) [59]. The analysis included antibiotics from five major categories: beta-lactams (penicillin, P 10 µg), cephalosporins (cephalothin, CN 30 µg; cefuroxime, CXM 30 µg; cefotaxime, CT 10 µg), glycopeptides (vancomycin, VA 30 µg), phenicols (chloramphenicol, C 30 µg), and tetracyclines (tetracycline, TE 30 µg). To perform the assay, 100 µL of each LAB culture was evenly spread onto MRS agar plates. After drying, antibiotic discs were placed on the agar surface. The plates were then incubated at 37 °C for 48 h. Following incubation, the diameters of the clear zones surrounding each disc were measured in millimeters to assess bacterial sensitivity. Based on the criteria established by the Clinical and Laboratory Standards Institute (CLSI) [60], the strains were categorized as susceptible (S) with inhibition zones ≥ 20 mm, intermediate (I) with inhibition zones of 10–20 mm, and resistant (R) if the zone measured ≤ 10 mm.

### 2.4. Preservation of LAB Isolates Through Freeze-Drying

LAB isolates were preserved through freeze-drying to ensure long-term viability and were applied following the protocol outlined by Diguță et al. [56]. LAB cultures were grown to the late exponential phase and harvested by centrifugation at 4000× *g* for 10 min at 4 °C. The pellet cells were resuspended in 5% maltodextrin as a cryoprotective medium to enhance cell survival. After freezing at −20 °C for at least 24 h, the samples were subjected to freeze-drying using a FreeZone 6 freeze dryer (Labconco, Kansas City, MO, USA). The resulting dried powders were stored in airtight containers at 4 °C until further use. The survival rate of the LAB strains following freeze-drying was determined using the following formula:

$$\%\;\mathrm{viability}=\left(\frac{\log\;\mathrm{CFUfd}\;}{\log\;\mathrm{CFUi}}\right)\times 100$$

where CFUi and CFUfd represent the colony-forming units before and after freeze-drying, respectively.

### 2.5. Evaluation of Postbiotic Properties of CFSs Derived from LAB Strains

#### 2.5.1. Preparation of Cell-Free Supernatants (CFSs) from Probiotic Bacterial Cultures

Fresh LAB cells were harvested by centrifugation at 10,000× *g* for 10 min at 4 °C using a Universal 320 R centrifuge (Hettich, Sérézin du Rhône, France). The resulting supernatant, rich in microbial metabolites, was filtered through 0.22 µm Millipore membranes (Sartorius, Göttingen, Germany), freeze-dried using the previous method, and stored at −20 °C until further use.

#### 2.5.2. Antimicrobial Activity of CFSs

##### Agar Well Diffusion Assay

The agar well diffusion technique was used to examine the antimicrobial activity of the CFSs from the LAB strains [61]. In 160 mm diameter Petri dishes, 2 mL of standardized bacterial suspensions (10^7^ CFU·mL^−1^) was evenly spread onto Mueller–Hinton agar (Sigma, Darmstadt, Germany). Once the agar was solidified, sterile pins (6 mm diameter) were used to punch wells. Each well was filled with 100 µL of CFS. To facilitate CFS diffusion, the plates were kept at 4 °C for 4 h, followed by incubation at 37 °C for 24 h. Antibacterial efficacy was determined by measuring the diameter of the inhibition zones (in millimeters) surrounding the wells using a ruler. The results were categorized as follows: no inhibition (<1 mm), weak inhibition (2–8 mm), moderate inhibition (8–16 mm), and strong inhibition (>16 mm).

##### MBC Evaluations

The antibacterial activity of the CFSs was assessed using the microdilution method, following guidelines provided by the Clinical and Laboratory Standards Institute (CLSI) [60]. Fresh bacterial cultures were diluted to a 0.5 McFarland standard (10^6^ CFU·mL^−1^). Freeze-dried CFS powders were resuspended in sterile distilled water at an initial concentration of 100 mg·mL^−1^ and filtered through sterile 0.22 µm membranes. A 100 µL aliquot of each CFS solution was added to the first tube in a serial dilution series. Twofold serial dilutions were prepared in Mueller–Hinton broth (Tulip Diagnostics (P) LTD., Verna, India), yielding final concentrations ranging from 100 to 1.57 mg·mL^−1^. Then, 1 µL of the standardized bacterial suspension (10^6^ CFU·mL^−1^) was added to each tube. The negative control consisted of uninoculated Mueller–Hinton broth, while the positive control contained only the pathogenic bacteria without CFS. After 24 h of incubation at 37 °C, 10 µL from each dilution was plated on Mueller–Hinton agar and incubated at 37 °C for another 24 h. The minimum bactericidal concentration (MBC) was defined as the lowest CFS concentration at which no visible bacterial growth was observed.

#### 2.5.3. Assessment of the Biochemical Profile

##### Total Polyphenol Content

The total phenolic content (TPC) of the CFS samples was quantified using the Folin–Ciocalteu colorimetric method, as outlined by Asadi et al. [62]. In summary, 20 µL of CFS was mixed with 110 µL of 10% Folin–Ciocalteu reagent and 70 µL of 20% sodium carbonate (Na_2_CO_3_) solution. The resulting mixture was incubated at room temperature in the dark for 30 min. Following incubation, absorbance was recorded at 765 nm. A calibration curve was prepared using gallic acid standards ranging from 0.10 to 200 μg·mL^−1^. The results are expressed as micrograms of gallic acid equivalents per milliliter of sample (μg GAE·mL^−1^).

##### Total Flavonoid Content

The total flavonoid content (TFC) of the CFS samples was measured using an aluminum chloride colorimetric assay, following the procedure outlined by Hamad et al. [63]. In this method, 100 µL of the CFS sample was mixed with 100 µL of 2% AlCl_3_ solution. The mixture was then incubated in the dark at room temperature for 30 min. After incubation, absorbance was read at 415 nm. A standard calibration curve was prepared using quercetin concentrations ranging from 0.10 to 200 μg·mL^−1^. The results are reported as micrograms of quercetin equivalents per milliliter of sample (μg QE·mL^−1^).

##### Assessment of Fourier Transform Infrared Spectroscopy of CFS Samples

Fourier transform infrared (FTIR) spectra of the CFS samples were obtained using a Tensor 27 FTIR spectrometer (Bruker Optik GmbH, Billerica, MA, USA) in the range of 4000–400 cm^−1^. The background was taken against the air using the same parameter settings.

#### 2.5.4. Antioxidant Activity by DPPH Assay

The antioxidant activity of the CFS samples was evaluated using a 2,2-diphenyl-1-picrylhydrazyl (DPPH) radical scavenging assay, following the protocol described by Qadi et al. [64]. Briefly, 100 µL of 150 µM DPPH solution was mixed with 50 µL of CFS and 50 µL of ultrapure water. The mixture was then incubated for 30 min in the dark at room temperature, and the absorbance of the mixture was measured at 517 nm. Subsequently, the absorbance measurements were applied to calculate the antioxidant activity, which is expressed as inhibition percent. Also, a Trolox calibration curve (0.10–125 μg·mL^−1^) was used, and the antioxidant activity of the CFS samples is expressed as μg Trolox equivalents per mL of sample (μg TE·mL^−1^).

### 2.6. Statistical Analysis

Statistical analyses were conducted using IBM SPSS Statistics version 30 (IBM Corp., Armonk, NY, USA). Each experiment was performed independently in triplicate (n = 3), and the results are expressed as mean values ± standard deviation. ANOVA and Friedman tests were used to compare samples for all parameters, alongside post hoc Tukey and Mann–Whitney tests, based on the normality of the distribution. All tests were two-sided, and the significance level was 5%.

GraphPad Prism Software version 10.4.1 (Boston, MA, USA) was used to assess the statistical significance for TPC, TFC, and antioxidant activity.

## 3. Results

### 3.1. Molecular Characterization of the LAB Isolates

The 16S rRNA gene sequencing elucidated the taxonomic identities of the 11 LAB strains, revealing ***Enterococcus faecium*** (strain MI120; NCBI accession number PV400812), ***Pediococcus pentosaceus*** (strains MI121–125; NCBI accession numbers PV400813–PV400817), and ***Pediococcus acidilactici*** (strains MI126–130; NCBI accession numbers PV400818–PV400822) (Table 1).

### 3.2. Probiotic Patterns of LAB Strains

#### 3.2.1. Resistance to Simulated Gastrointestinal Conditions

**Figure 1:**
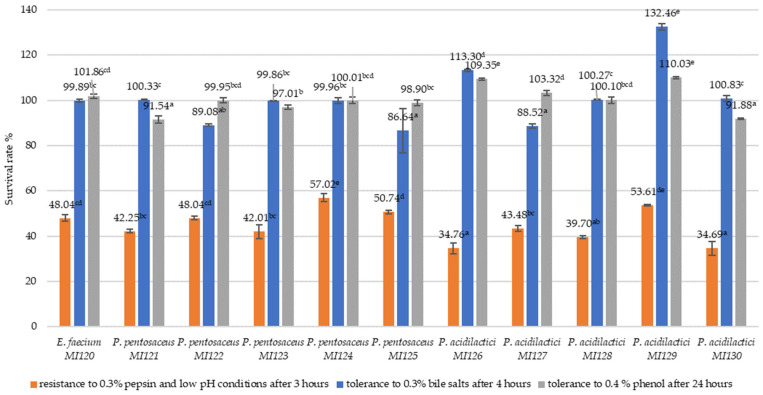
Survival rate (%) of LAB strains under simulated gastrointestinal conditions. Data represent mean ± SD (n = 3). Different letters denote significant differences between strains (*p* < 0.05, one-way ANOVA, Tukey B test).

The LAB strains were evaluated for their ability to endure simulated gastrointestinal conditions (Figure 1).

Under simulated gastric conditions (0.3% pepsin, low pH, and 3 h), the survival rates varied across strains, ranging from 34.69% to 57.02%. The highest resistance was observed to *P. pentosaceus* MI124 (57.02 ± 1.84%), followed by *P. acidilactici* MI129 (53.61 ± 0.31%) and *P. pentosaceus* MI125 (50.74 ± 0.84%). *E. faecium* MI120 exhibited a survival rate of 48.04 ± 1.38% under pepsin and low-pH stress (Figure 1).

All *Pediococcus* strains showed high survival under 0.3% bile salt conditions after 4 h, with *P. acidilactici* MI129 exhibiting the highest bile salt tolerance (132.46 ± 1.32%), followed by *P. acidilactici* MI126 (113.30 ± 0.53%) and *P. acidilactici* MI130 (100.83 ± 1.19%). Among the *P. pentosaceus* strains, MI121 showed the most excellent tolerance (100.33 ± 0.23%) (Figure 1).

All LAB strains evaluated demonstrated a strong ability to tolerate 0.4% phenol, exceeding 91%, confirming their function under harsh conditions. *P. acidilactici* MI129 showed the highest phenol tolerance (110.03 ± 0.46%), followed by MI126 (109.35 ± 0.46%) and MI127 (103.32 ± 1.13%). Among the *P. pentosaceus* strains, MI124 displayed high survival rates (100.01 ± 1.33%), while *E. faecium* MI120 also demonstrated significant phenol resistance (101.86 ± 0.98%) (Figure 1).

#### 3.2.2. Cell Surface Properties of LAB Strains

Cell surface characteristics are essential functional traits of probiotic strains, as they reflect the ability of microorganisms to adhere to intestinal epithelial surfaces and establish colonization.

Among the evaluated LAB strains, *P. pentosaceus* MI124 exhibited the highest auto-aggregation ability (81.88 ± 1.01%), followed closely by *P. acidilactici* MI127 (80.61 ± 1.51%), MI126 (78.52 ± 0.70%), and MI128 (78.50 ± 0.58%). All of these demonstrated strong aggregation potential. In contrast, *E. faecium* MI120 (39.49 ± 1.72%) showed the lowest auto-aggregation capacities, suggesting comparatively weaker adhesive properties and a lower colonization potential (Table 2).

The results revealed strain-dependent variability in hydrophobic interactions. Among the tested strains, *P. acidilactici* MI127 exhibited the highest hydrophobicity with xylene (55.04 ± 0.97%), followed by *P. pentosaceus* MI122 (31.84 ± 1.69%) and *P. acidilactici* MI129 (31.17 ± 1.39%). With hexane, *P. pentosaceus* MI121 had the highest hydrophobicity (24.11 ± 1.68%), followed by *P. pentosaceus* MI123 (22.19 ± 0.46%), while *P. pentosaceus* MI125 (3.98 ± 1.25%) and *P. acidilactici* MI126 (2.99 ± 0.47%) showed the lowest values (Table 2).

#### 3.2.3. Enzymatic Profile

All LAB isolates lacked amylase and cellulase activities, consistent with typical lactic acid bacteria that do not rely on starch or cellulose degradation. Additionally, all strains were catalase-negative and gelatinase-negative, enhancing their safety profile, a trait frequently sought after in probiotic strains (Table 3).

The data also demonstrate that all strains showed a consistent dual phytase and protease activity profile, thereby supporting their functional versatility (Table 3).

The APIZYM analysis revealed distinct multi-enzyme profiles among the LAB strains, highlighting shared functional capabilities and specific patterns for each strain (Table 4).

*E. faecium* MI120 demonstrated a wide range of enzymatic activities, including weak-to-moderate lipase activity, leucine arylamidase activity, and strong acid phosphatase activity, alongside exclusive alkaline phosphatase expression. This profile suggests that the strain has the capacity for vigorous lipid and phosphate metabolism.

The *P. pentosaceus* group demonstrated a more uniform enzymatic profile, combining weak but consistent lipolytic activity with a pronounced saccharolytic potential. Enzymes such as β-galactosidase, β-glucosidase, and N-acetyl-β-glucosaminidase were commonly expressed across these strains. Among them, MI124 displayed the most complex enzymatic repertoire, encompassing esterase and lipase activities (C4, C8, and C14), multiple glycoside hydrolases (β-glucosidase, β-galactosidase, β-glucuronidase, N-acetyl-β-glucosaminidase, and α-mannosidase), proteases (including cystine, valine, leucine arylamidase, and α-chymotrypsin), and phosphatases (acid phosphatase and Naftol-AS-BI-phosphohydrolase).

*P. acidilactici* consistently exhibited strong leucine and valine arylamidase activities, whereas trypsin activity was uniquely found in MI127 to MI129, suggesting broader protease functionality in these strains. Except for in the MI129 strain, lipase and esterase activities (C4–C14) were consistently observed. All isolates also showed positive results for Naftol-AS-BI-phosphohydrolase and acid phosphatase, except for MI130, which lacked acid phosphatase. Enzymes involved in carbohydrate metabolism were strain-dependent: MI126 and MI127 exhibited moderate β-galactosidase and β-glucosidase activities, while MI130 displayed a more extensive saccharolytic profile, including high levels of β-glucosidase and N-acetyl-β-glucosaminidase, along with moderate β-galactosidase activity.

#### 3.2.4. Safety Traits of LAB Strains

The *E. faecium* MI120 and *P. acidilactici* strains demonstrated susceptibility to tetracycline and chloramphenicol. The *P. pentosaceus* strains showed intermediate responses to tetracycline and sensitivity to chloramphenicol. All strains were resistant to cefotaxime, cefuroxime, cephalothin, and penicillin, with *E. faecium* MI120 being the only strain susceptible to vancomycin (Table 5).

Furthermore, all strains exhibited γ-hemolysis, supporting their classification as safe for use (Table 5).

#### 3.2.5. Freeze-Drying as a Method for Long-Term Preservation of LAB Strains

**Figure 2:**
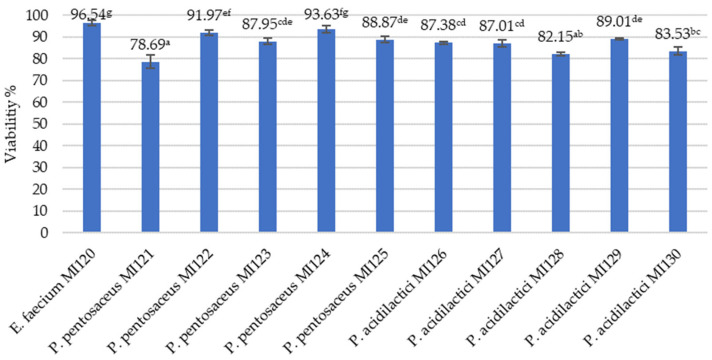
Viability percentages of freeze-dried LAB strains. Data represent mean ± SD (n = 3). Different letters denote significant differences between strains (*p* < 0.05, one-way ANOVA, Tukey B test).

*E. faecium* M120 had the highest survival rate (96.54 ± 1.16%), indicating its strong resistance to freeze-drying. The *P. pentosaceus* MI124 and MI122 strains also exhibited high survival rates (exceeding 91%), making them potential candidates for long-term probiotic formulations (Figure 2).

### 3.3. Postbiotic Profiles of LAB Strains

#### 3.3.1. Antimicrobial Patterns of LAB Strain

The antimicrobial activity of the LAB strains was evaluated using the agar well diffusion method. A panel of Gram-positive and Gram-negative pathogenic bacteria was used to assess the broad-spectrum efficacy of the strains (Table 6). The *P. pentosaceus* and *P. acidilactici* strains exhibited notably stronger antibacterial activity compared to *E. faecium* MI120, particularly against Gram-positive bacteria. Strong inhibition was observed for *P. pentosaceus* MI121, MI122, and MI124, as well as for *P. acidilactici* MI127 and MI129, especially against *Staphylococcus aureus* (ATCC 33592 and 6538), *Staphylococcus epidermidis* (ATCC 551625 and 12228), and *Proteus vulgaris* ATCC 13315. These findings support these strains’ robust and broad-spectrum antimicrobial potential, particularly against clinically relevant Gram-positive pathogens.

All LAB strains exhibited a moderate-to-strong inhibition of *Bacillus cereus, Listeria monocytogenes,* and *Escherichia coli*, indicating their potential as effective biocontrol agents against common foodborne pathogens.

Compared to the *Pediococcus* strains, *E. faecium* MI120 demonstrated lower antimicrobial efficacy, as evidenced by weak-to-moderate inhibition against all tested pathogens.

However, *P. aeruginosa*, *S. marcescens*, *K. kristinae*, and *R. equi* were generally more resistant, with most LAB strains showing weak or moderate inhibition.

The MBC values of the CFSs from the LAB strains against a panel of pathogens varied from 3.13 to 100 mg·mL^−1^, indicating broad-spectrum bactericidal activity (Table 7).

The results are presented as the mean ± standard deviation (SD) of three independent experiments, with significant differences observed across all comparisons (*p* < 0.05, Mann–Whitney test). Significant differences in antibacterial effectiveness were observed among the LAB strains and target pathogens. Most *Pediococcus* strains showed strong activities (3.13–12.5 mg·mL^−1^) against *P. vulgaris*, *P. aeruginosa,* and *S. epidermidis.* Foodborne pathogens such as *Bacillus cereus*, *Escherichia coli*, *Listeria monocytogenes*, *Salmonella* Typhimurium, and *Salmonella* Enteritidis exhibited strain-specific susceptibility to *Pediococcus*-derived CFSs, with MBCs ranging from 6.25 to 50 mg·mL^−1^. Notably, *P. pentosaceus* MI124 and *P. acidilactici* MI128–MI130 showed enhanced bactericidal effects against these pathogens. In contrast, *E. faecium* MI120 exhibited the lowest efficacy observed against *B. cereus, E. coli*, and *S.* Typhimurium (100 mg·mL^−1^). *S. aureus* was particularly sensitive, with low MBCs (6.25–12.5 mg·mL^−1^) for most *Pediococcus* strains. *R. equi* exhibited moderate resistance, with MBC values ranging from 12.5 to 50 mg·mL^−1^, while *S. marcescens* was more susceptible, with MBCs between 12.5 and 25 mg·mL^−1^. *Kocuria kristinae* MI20 exhibited variable susceptibility to LAB-derived CFSs, with MBC values ranging from 3.13 to 100 mg·mL^−1^, with the lowest being found for the CFS derived from *E. faecium* MI120.

#### 3.3.2. Biochemical Profile

The evaluation of the total phenolic content (TPC) and total flavonoid content (TFC) in CFSs revealed significant inter-strain variation in phenolic profiles (Table 8). Among all tested strains, *P. pentosaceus* MI121 recorded the highest TPC (162.18 ± 2.05 μg·mL^−1^), closely followed by *E. faecium* MI120 (159.99 ± 1.35 μg·mL^−1^), indicating that these strains possess an enhanced metabolic capacity for producing or secreting phenolic compounds.

The TFC showed no notable differences among most strains, ranging from approximately 20 to 22 μg·mL^−1^. However, *P. pentosaceus* MI123 demonstrated a significantly lower TFC (17.57 ± 0.17 μg·mL^−1^), suggesting potential strain-specific limitations in flavonoid biosynthesis or secretion.

**Figure 3:**
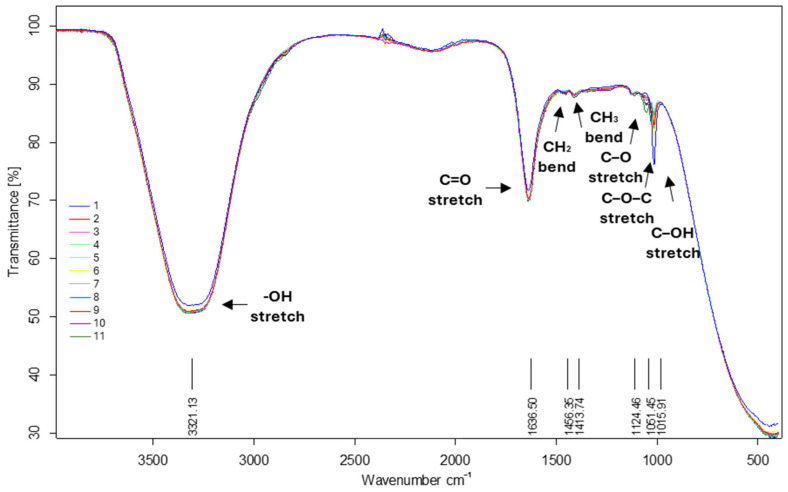
FTIR spectra of CFS samples. 1—*E. faecium* MI120, 2—*P. pentosaceus* MI121, 3—*P. pentosaceus* MI122, 4—*P. pentosaceus* MI123, 5—*P. pentosaceus* MI124, 6—*P. pentosaceus* MI125, 7—*P. acidilactici* MI126, 8—*P. acidilactici* MI127, 9—*P. acidilactici* MI128, 10—*P. acidilactici* MI129, 11—*P. acidilactici* MI130.

Fourier transform infrared (FTIR) spectroscopy further supported the biochemical profile of the CFSs, as shown in Figure 3. The notable absorption peaks observed included 3321 cm^−1^ (stretching vibrations of the -OH group), 1636 cm^−1^ (stretching vibrations of the C=O amide group), 1456 cm^−1^ (bending vibrations of the CH_2_ group), 1413 cm^−1^ (bending vibrations of the CH_3_ group), 1124 (stretching vibrations of the C–O group, a characteristic peak of lactic acid), 1051 cm^−1^ (stretching vibrations of the C-O-C group), and 1015 cm^−1^ (stretching vibrations of C-OH groups).

#### 3.3.3. Antioxidant Activity

All CFSs showed significant DPPH radical scavenging activity, with inhibition percentages of 66.05% and 75.19% (Table 9). *E. faecium* MI120 exhibited the highest inhibition (75.19 ± 0.37%), corresponding with its high TPC value, suggesting a positive relationship between the phenolic content and antioxidant activity. Moreover, *P. pentosaceus* MI124 had the lowest values for both inhibition (66.05 ± 2.35%) and the TPC (151.95 ± 1.57 μg.mL^−1^), aligning with these findings.

The IC_50_ values, expressed as dilution factors, ranged from 1/4 to 1/12, with *E. faecium* MI120 (1/12) and *P. acidilactici* MI129 and MI130 (1/11) demonstrating the most potent antioxidant effects. The Trolox equivalent (TE) values further supported these findings, with MI120 (21.37 ± 0.10 μg·mL^−1^) and MI130 (20.53 ± 0.15 μg·mL^−1^) recording the highest antioxidant equivalents.

## 4. Discussion

Recently, increasing research has demonstrated that other LAB species besides *Lactobacillus* and *Bifidobacterium* have garnered significant attention as probiotics for their potential roles in developing therapeutic agents, natural antimicrobials, and bio-based food preservatives [11,12,13,14,15,16,17]. The results of 16S rRNA sequencing identified our isolates as *Enterococcus faecium* (one strain), *Pediococcus acidilactici* (five strains), and *Pediococcus pentosaceus* (five strains). The most recent update of the European Food Safety Authority’s (EFSA) Qualified Presumption of Safety (QPS) list includes several *Pediococcus* strains, reflecting their acceptance under EFSA’s framework for assessing the safety of microorganisms used in food and feed production [50]. However, Mantzios et al. [65] reported a case of endocarditis following transcatheter aortic valve implantation in a patient with pre-existing health conditions; they faced methodological limitations regarding strain identification. Importantly, no findings from the reviewed literature warranted any revision of the current QPS status of the *Pediococcus* species included in the list [50]. Numerous studies have highlighted the probiotic potential and safety profiles of *P. acidilactici* and *P. pentosaceus*, supporting their suitability for use as probiotic candidates [12,24,55,64,65,66,67,68,69]. Unlike other LAB genera, *Enterococcus* species have not yet obtained QPS status. Nevertheless, various researchers have reported *Enterococcus* species’ probiotic attributes and safety aspects, indicating their potential use as probiotics [67,70,71,72,73,74].

LAB strains must meet specific safety and functional standards to qualify for probiotic use; therefore, a thorough characterization of our strains is essential for identifying the most suitable ones.

In the present study, simulated gastric and bile tolerance tests revealed strain-dependent variability. Among the tested LAB strains, *P. pentosaceus* MI124 and *P. acidilactici* MI129 showed the most promising overall performance. These strains demonstrated strong resistance to pepsin and low pH (57.02% and 53.61%, respectively) and high bile salt tolerance (99.96% and 132.46%, respectively), underscoring their potential as probiotics. These strains also exhibited superior resistance to phenol, a toxic by-product in the gut, with survival rates exceeding 100%. Their survival under gastrointestinal stress demonstrates their ability to remain viable while passing through the stomach and small intestine, which is essential for providing beneficial effects in the host.

Auto-aggregation is often linked to the ability of probiotic strains to form biofilms [75,76], which is essential for inhibiting pathogen adhesion to the intestinal mucosa. In the current study, the observed auto-aggregation levels among the tested *P. acidilactici* strains fell within the range of previously reported values. For instance, *P. acidilactici* Kp10 demonstrated 35.2% auto-aggregation [66], while *P. acidilactici* SMVDUDB2 showed a significantly higher aggregation rate of 77.68% [68]. These findings support the notion that auto-aggregation is a strain-specific trait, and they emphasize the importance of evaluating individual isolates when assessing probiotic functionality.

According to Tyfa et al. [77], bacterial strains can be categorized based on their affinity to hydrocarbons: those with hydrophobicity values above 50% are considered strongly hydrophobic, those with values between 20% and 50% have moderate hydrophobicity, and those with values below 20% are classified as hydrophilic. In our study, *P. acidilactici* MI129 (hexane: 17.54 ± 0.88%; xylene: 31.17 ± 1.39%) and *P. pentosaceus* MI123 (hexane: 22.19 ± 0.46%; xylene: 21.05 ± 1.04%) exhibited, for the most part, a moderate affinity for both solvents, demonstrating balanced surface properties that enhance their suitability for probiotic applications.

The enzymatic profiling conducted in this study revealed distinct patterns that varied between LAB species, illustrating the metabolic heterogeneity across the *Pediococcus* and *Enterococcus* genera. Enzyme expression was influenced by both species and individual strain characteristics, reflecting diverse functional capabilities. In agreement with the findings of Coulibaly et al. [12], none of the LAB strains in this study exhibited amylase or cellulase activity. Nevertheless, their expression of phytase and protease reflects promising enzymatic characteristics that can enhance dietary nutrient absorption and positively influence host health. The *P. pentosaceus* strains generally displayed pronounced activity in both proteolytic and saccharolytic pathways. Among them, MI124 was particularly notable for its extensive enzymatic activity, encompassing a range of hydrolases, esterases, proteases, and phosphatases. In comparison, the *P. acidilactici* strains exhibited more diverse enzymatic profiles, especially in lipid and carbohydrate metabolism, with MI130 demonstrating the most comprehensive saccharolytic enzyme expression. Meanwhile, *E. faecium* MI120 showed a unique enzymatic signature, characterized by prominent lipase and phosphatase activities yet lacking glycoside hydrolase expression. *Enterococcus* strains isolated from traditional Greek Graviera and Galotyri PDO cheeses demonstrated strain-dependent enzyme activity patterns [27]. These isolates generally exhibited moderate alkaline phosphatase levels and moderate-to-high activities for esterase (C4) and esterase/lipase (C8). Additionally, acid phosphatase and napthol-AS-BI-phosphohydrolase activities were present, while amino acid arylamidase reactions, including leucine, valine, and cystine, ranged from moderate to strong [27]. These results underscore the species-dependent nature of the enzyme activity within LAB, and they emphasize the importance of detailed strain-level profiling when selecting candidates for specific probiotic or postbiotic purposes. Notably, the wide-ranging enzymatic profiles of *P. pentosaceus* MI124 and *P. acidilactici* MI130 highlight their potential as versatile and practical components in functional health-promoting formulations.

In recent years, the increased awareness of the health-promoting properties of probiotics and postbiotics has led to the widespread application of drying technologies to stabilize final products [78,79,80]. Among these, freeze-drying has emerged as a preferred method for producing stable powdered formulations for long-term storage, as it helps to preserve the viability and bioactivity of microbial strains [81]. Selecting suitable cryoprotectants can significantly enhance the survival rate of probiotics during freeze-drying [78,79,80,81]. Our results demonstrated that *E. faecium* MI120 and the *P. pentosaceus* strains MI124 and MI122 maintained high viability after freeze-drying (96.54%, 93.63%, and 91.97%, respectively), aligning with the outcomes reported by Coulibaly et al. [12] and Diguță et al. [56], as well as underscoring their potential as strong candidates for formulation into long-lasting probiotic or postbiotic supplements.

Therefore, it is crucial to evaluate their antibiotic susceptibility to ensure safety before their application as probiotic candidates. All strains exhibited intermediate or susceptible responses to tetracycline and chloramphenicol, suggesting a low risk of resistance gene transfer for these antibiotics, which aligns with EFSA safety guidelines. Notably, *E. faecium* MI120 was the only strain susceptible to vancomycin, whereas all *Pediococcus* strains resisted clinically used β-lactam antibiotics, including cefotaxime, cefuroxime, cephalothin, and penicillin. This antibiotic resistance presents a potential safety issue, underscoring the importance of supplementary assessments of resistance determinants prior to their use in probiotic formulations [82]. Moreover, all strains were γ-hemolytic, catalase-negative, and gelatinase-negative, reinforcing their safety profile. The absence of these enzymes supports the notion that the tested strains are non-pathogenic and, therefore, suitable for probiotic application, following FAO/WHO safety guidelines [83]. Our results align with the data presented in the work of Coulibaly et al. [12], Diguță et al. [56], and Fugaban et al. [74].

Considering the risk of the horizontal gene transfer of antibiotic resistance traits from probiotics to harmful bacteria [82,83], postbiotics may provide a safer alternative for vulnerable groups, such as immunocompromised patients and those with a compromised gastrointestinal mucosa.

Overall, the strong antimicrobial profiles of the *Pediococcus* strains, particularly *P. pentosaceus* MI124 and *P. acidilactici* MI129, reflected by low MBC values, suggest their effectiveness as postbiotic candidates against various foodborne and clinical pathogens. Our results are consistent with those of Diguță et al. [56], who investigated the antimicrobial potential of *P. pentosaceus* and *P. acidilactici* strains isolated from the Kombucha consortium. The *E. faecium* RC001, *P. acidilactici* RC004, and *P. pentosaceus* RC007 strains demonstrated strong antimicrobial activity against multidrug-resistant *Salmonella* spp. and *Escherichia coli* pathogens [84]. A study involving *P. pentosaceus* ELAB 60WB, isolated from fermented cherry tomatoes, reported strong antagonistic effects against *S. typhimurium* ATCC 14,028 (26 mm), *S. pyogenes* ATCC 19,615 (21 mm), *K. pneumoniae* ATCC 13,883 (16 mm), and *S. aureus* ATCC 29,213 (14 mm), while no inhibition was observed against *E. coli* ATCC 25,922 [85]. In a previous study, the CFS of *Pediococcus pentosaceus* PMY2 exhibited strong antimicrobial activity, with MIC/MBC values of 0.31/0.63 mg·mL^−1^ against *S. aureus*, 0.31/2.5 mg·mL^−1^ against MRSA, 0.16/0.31 mg·mL^−1^ against *P. aeruginosa* (including the MDR strain), and 0.16/0.63 mg·mL^−1^ against *E. coli* (including the MDR strain) [69]. The fermentation supernatant of *Enterococcus faecium* AB157 exhibited notable antibacterial activity, producing inhibition zones of 13.52 ± 0.16 mm against *Pseudomonas aeruginosa* and 11.14 ± 0.09 mm against *Vibrio parahaemolyticus*, while no inhibition was observed against *Staphylococcus aureus* [86].

Our findings demonstrate that the CFSs obtained from LAB strains possess antioxidant and antimicrobial properties, underlining their potential as rich sources of phenolic compounds contributing to various health-promoting effects. The total phenolic content (TPC) ranged from 151.77 ± 2.11 to 162.18 ± 2.05 μg mL^−1^. As noted by İncili et al. [87], phenolic acids are multifunctional, acting as potent antioxidants and agents with antimicrobial activity. In contrast, the total flavonoid content (TFC) remained relatively constant and low across our strains, suggesting limited strain-dependent variation in flavonoid biosynthesis. FTIR characteristic absorption peaks confirmed the presence of functional groups associated with phenolic compounds, organic acids, and other bioactive metabolites. The presence of these groups highlights the biochemical complexity of the CFSs and reinforces their potential as sources of functional bioactive molecules. The overall spectral profiles of the strains were similar, indicating the presence of shared classes of metabolites. However, there were notable differences in the intensities of the absorption bands between specific CFSs. *E. faecium* MI120 displayed significantly higher intensities in the C–O, C–O–C, and C–OH regions (~1124–1015 cm^−1^). These differences suggest a greater accumulation of lactic acid and related hydroxyl-containing metabolites, aligning with the strain’s metabolic output. In contrast, samples derived from the *P. pentosaceus* strains (MI121–MI125) showed more moderate signals in these regions. This finding indicates a lower lactic acid concentration or a different balance of metabolic by-products in these samples. Nevertheless, they exhibited higher intensities in the broad O–H stretching region (~3321 cm⁻^1^) and the C=O stretching region (~1636 cm⁻^1^). The characteristic absorption peaks of the CFS samples matched those in the existing literature [88,89,90,91].

Among the well-recognized benefits of probiotic strains is their ability to counteract oxidative stress, a significant factor in cellular injury and chronic illnesses. Lactic acid bacteria grown in MRS medium produce multiple bioactive metabolites contributing to their antioxidant effects. Following the findings of the aforementioned studies, the *E. faecium* MI120 and *P. pentosaceus* MI121 strains, along with other LAB strains investigated in our research, exhibit remarkable antioxidant properties linked to their phenolic profiles. For example, *P. pentosaceus* MYU 759 [91] and *P. pentosaceus* M41 [92] exhibited notable hydroxyl radical scavenging activity, attributed to the production of acidic exopolysaccharides (EPSs). Additionally, several studies have highlighted the antioxidant potential of cell-free supernatants (CFSs) from *Pediococcus* strains. Notably, investigations by Coulibaly et al. [12], Diguță et al. [56], Rosales Cavaglieri [84], and Łepecka et al. [93] demonstrated significant antioxidant activity, reinforcing the bioactive potential of the postbiotic compounds produced by LABs.

The findings of this study highlight the significant potential of the selected LAB strains as alternative biocontrol agents and sources of bioactive postbiotics for enhancing food safety and promoting health [51,52,53,54,94,95,96]. However, the successful application of these strains depends on carefully selecting candidates that meet stringent safety and efficacy standards, supported by robust clinical evidence to validate their health benefits [94,95,96]. Future research should aim to confirm the absence of genes associated with virulence, antibiotic resistance, or toxin production, as well as evaluate the risk of horizontal gene transfer that could have public health implications [95,96,97]. Additionally, comprehensive analytical studies, such as HPLC, are needed to identify the specific bioactive compounds in CFSs and explore the potential synergistic effects between multiple CFSs or naturally occurring bioactive substances [98,99,100,101,102]. It is also crucial to assess the stability of these LAB strains under industrial processing conditions [98,99]. These insights lay a strong foundation for developing innovative probiotic and postbiotic formulations for practical application in the food and nutraceutical industries to reduce dependence on traditional antibiotics [51,52,53,54,94,95,96,102,103,104,105]. In the study by Jalali et al. [102], the potential of postbiotics derived from Lactobacilli as natural antimicrobial agents was highlighted. They demonstrated their effectiveness in inhibiting the growth of common foodborne pathogens, *Escherichia coli* and *Staphylococcus aureus*, on meat products, contributing to an extended shelf life. In another study, Viazis et al. [105] conducted a randomized controlled trial assessing the efficacy of combining probiotics (*Lactobacillus acidophilus LA-5, Lactiplantibacillus plantarum, Bifidobacterium lactis BB-12*, and *Saccharomyces boulardii*) with four antibiotics (amoxicillin, clarithromycin, metronidazole, and omeprazole) for the treatment of *Helicobacter pylori* infection. The trial reported a 92% eradication rate of *H. pylori* in the probiotic group, compared to 86.8% in the control group.

## 5. Conclusions

This study identified eleven LAB strains through 16S rRNA gene sequencing, including *Enterococcus faecium, Pediococcus acidilactici,* and *Pediococcus pentosaceus*. A comprehensive series of functional evaluations revealed substantial inter-strain variation in probiotic and postbiotic characteristics, thereby substantiating the strain-dependent nature of LAB bioactivity. Among the isolates evaluated, *P. pentosaceus* MI124 and *P. acidilactici* MI129 exhibited remarkable tolerance to simulated gastrointestinal conditions, robust phenol resistance, and significant survivability following freeze-drying. A surface property analysis revealed elevated auto-aggregation for *P. pentosaceus* MI124 and *P. acidilactici* MI127, as well as hydrophobicity in *P. pentosaceus* MI123 and *P. acidilactici* MI129, suggesting considerable potential for adhesion to the intestinal mucosa. Among the LAB strains, MI124 demonstrated the broadest spectrum of enzymatic activity. Safety evaluations confirmed that all strains were non-hemolytic, gelatinase-negative, and catalase-negative and exhibited varying antibiotic susceptibility patterns.

Postbiotic evaluations indicated broad-spectrum antimicrobial activity, particularly in *P. pentosaceus* MI124 and *P. acidilactici* MI129, with low MBCs against 14 pathogenic bacteria. A biochemical analysis was conducted to ascertain the presence of bioactive compounds. A positive correlation was established between the TPC and antioxidant capacity, supported by DPPH scavenging activity, IC50 values, and Trolox equivalents.

Overall, *P. pentosaceus* MI124 and *P. acidilactici* MI129 emerged as the most promising multifunctional candidates, combining excellent probiotic characteristics with strong postbiotic efficacy. Future research will focus on optimizing fermentation parameters, scaling up production processes, and assessing the stability and cost-effectiveness of these LAB strains and their postbiotic formulations. These strains show significant potential for development as functional food additives, nutraceuticals, or natural biopreservatives, offering promising opportunities for enhancing food safety and public health.

## Figures and Tables

**Table 1:** The accession numbers of the LAB strains used in this study.

LAB Strains	Codes	Accession Number
*Enterococcus faecium*	MI120	PV400812
*Pediococcus pentosaceus*	MI121	PV400813
	MI122	PV400814
	MI123	PV400815
	MI124	PV400816
	MI125	PV400817
*Pediococcus acidilactici*	MI126	PV400818
	MI127	PV400819
	MI128	PV400820
	MI129	PV400821
	MI130	PV400822

**Table 2:** Auto-aggregation and hydrophobicity (%) of LAB strains.

LAB Strains	Codes	Auto-Aggregation %	Hydrophobicity (H) (%)	
			Hexane	Xylene
*E. faecium*	MI120	39.49 ± 1.72 ^a^	15.39 ± 1.12 ^f^	14.54 ± 2.15 ^b^
*P. pentosaceus*	MI121	70.53 ± 0.93 ^d^	24.11 ± 1.68 ^g^	9.76 ± 0.96 ^b^
	MI122	58.19 ± 5.24 ^b^	7.41 ± 1.22 ^bc^	31.84 ± 1.69 ^d^
	MI123	63.25 ± 0.62 ^c^	22.19 ± 0.46 ^g^	21.05 ± 1.04 ^c^
	MI124	81.88 ± 1.01 ^d^	11.23 ± 0.82 ^e^	22.55 ± 4.02 ^c^
	MI125	69.00 ± 1.13 ^cd^	3.98 ± 1.25 ^a^	22.94 ± 0.69 ^c^
*P. acidilactici*	MI126	78.52 ± 0.70 ^d^	2.99 ± 0.47 ^a^	25.04 ± 2.22 ^c^
	MI127	80.61 ± 1.51 ^d^	5.01 ± 0.91 ^ab^	55.04 ± 0.97 ^e^
	MI128	78.50 ± 0.58	8.07 ± 0.60 ^cd^	10.05 ± 0.97 ^b^
	MI129	41.77 ± 2.77 ^a^	17.54 ± 0.88 ^f^	31.17 ± 1.39 ^d^
	MI130	54.16 ± 0.74 ^b^	10.33 ± 0.84 ^de^	3.82 ± 1.12 ^a^

Data represent mean ± SD (n = 3). Different letters denote significant differences between strains (*p* < 0.05, one-way ANOVA, Tukey B test).

**Table 3:** Hydrolytic enzyme profile of the LAB isolates.

LAB Strains	Codes	Enzymatic Activities					
		Amylases	Cellulases	Catalase	Gelatinase	Phytase	Proteases
*E. faecium*	MI120	−	−	−	−	+	+
*P. pentosaceus*	MI121	−	−	−	−	+	+
	MI122	−	−	−	−	+	+
	MI123	−	−	−	−	+	+
	MI124	−	−	−	−	+	+
	MI125	−	−	−	−	+	+
*P. acidilactici*	MI126	−	−	−	−	+	+
	MI127	−	−	−	−	+	+
	MI128	−	−	−	−	+	+
	MI129	−	−	−	−	+	+
	MI130	−	−	−	−	+	+

Legend: no activity (−); detection activity (+).

**Table 4:** APIZYM profile of LAB strains.

Enzymes	*E. faecium*	*P. pentosaceus* Strains					*P. acidilactici* Strains				
	MI120	MI121	MI122	MI123	MI124	MI125	MI126	MI127	MI128	MI129	MI130
Alkaline phosphatase	+	−	−	−	−	−	−	−	−	−	−
Esterase (C4)	++	+	+	+	+	+	+	+	+	−	+
Esterase lipase (C8)	++	+	+	+	+	+	+	+	+	−	+
Lipase (C14)	+	+	+	+	+	+	+	+	+	−	+
Leucine arylamidase	++	+++	+++	+++	+++	+++	+++	+++	+++	+++	+++
Valine arylamidase	−	+++	+++	+++	+++	+++	+++	+++	+++	+++	+++
Cystine arylamidase	−	+	+	−	+	−	+	+	+	+	−
Trypsin	−	−	−	−	−	−	−	+	+	+	−
α-chymotrypsin	−	−	−	−	+	−	−	−	−	−	−
Acid phosphatase	+++	+	+	+	+	−	+	+	+	+	−
Naftol-AS-BI-phosphohydrolase	+	+	++	++	++	+++	+	++	++	++	+
α-galactosidase	−	−	−	+	−	−	−	−	−	−	−
β-galactosidase	−	+++	+++	+++	+++	+++	+	+	−	−	++
β-glucuronidase	−	−	−	−	+	−	−	−	−		−
α-glucosidase	−	−	−	−	−	−	−	−	−	−	−
β-glucosidase	−	+++	+++	+++	+++	+++	+	+	−	−	+++
N-acetyl-β-glucosaminidase	−	+++	+++	+++	+++	+++	++	−	−	−	+++
α-mannosidase	−	−	−	−	+	−	−	++	+	−	−

Legend: no activity (−); weak activity (+); moderate activity (++); strong activity (+++).

**Table 5:** Evaluation of safety characteristics of LAB strains.

LAB Strains	Codes	Antibiotics Susceptibility							Hemolytic Activity
		TE30	C30	CT10	VA30	CXM30	CN30	P10	
*E. faecium*	MI120	S	S	R	S	R	R	R	γ
*P. pentosaceus*	MI121	I	S	R	R	R	R	R	γ
	MI122	I	S	R	R	R	R	R	γ
	MI123	I	S	R	R	R	R	R	γ
	MI124	I	S	R	R	R	R	R	γ
	MI125	I	S	R	R	R	R	R	γ
*P. acidilactici*	MI126	S	S	R	R	R	R	R	γ
	MI127	S	S	R	R	R	R	R	γ
	MI128	S	S	R	R	R	R	R	γ
	MI129	S	S	R	R	R	R	R	γ
	MI130	S	S	R	R	R	R	R	γ

S: susceptible (≥20 mm); I: intermediate (10–20 mm); R: resistant (<10 mm). P10 (penicillin), CN30 (cephalothin), CXM30 (cefuroxime), CT10 (cefotaxime), TE30 (tetracycline), C30 (chloramphenicol); VA30 (vancomycin); γ (Gamma) hemolysis.

**Table 6:** In vitro antibacterial activities of CFSs derived from LAB strains.

Pathogenic Bacteria	*E. faecium*	*Pediococcus pentosaceus* Strains					*Pediococcus acidilactici* Strains				
	MI120	MI121	MI122	MI123	MI124	MI125	MI126	MI127	MI128	MI129	MI130
*B. cereus* ATCC 11778	+	++	++	++	+++	++	++	++	++	++	++
*E. coli* ATCC 8739	+	++	++	++	+++	++	++	+++	+++	+++	++
*K. kristianae* MI 20	+	++	++	++	++	++	++	++	++	++	++
*L. monocytogenes* ATCC 13932	++	++	++	++	++	+++	++	++	++	++	++
*P. aeruginosa* ATCC 15442	+	+	++	++	++	++	+	++	+	+	+
*R. equi* ATCC 6939	++	++	++	++	++	++	++	++	+	+	++
*P. vulgaris* ATCC 13315	++	+++	+++	++	++	+++	+++	+++	+++	++	++
*S.* Typhimurium ATCC 14028	+	++	++	++	++	++	++	++	++	++	++
*S.* Enteretidis ATCC 13076	++	++	++	++	++	++	++	++	++	++	++
*S. marcescens* ATCC 14756	+	+	++	++	++	++	++	++	++	++	+
*S. aureus* ATCC 33592	++	+++	+++	+++	+++	+++	+++	+++	+++	+++	+++
*S. aureus* ATCC 6538	++	+++	+++	++	+++	+++	+++	+++	++	+++	+++
*S. epidermidis* ATCC 551625	++	++	+++	+++	+++	++	++	+++	++	+++	++
*S. epidermidis* ATCC 12228	++	++	+++	+++	+++	+++	++	+++	++	+++	++

Weak inhibition (+), 1 mm–10 mm; moderate inhibition (++), 10 mm–20 mm; strong inhibition (+++) >20 mm.

**Table 7:** Minimum bactericidal concentration (MBC) values (mg·mL^−1^) of CFSs against pathogenic bacteria.

Pathogenic Bacteria	*E. faecium*	*P. pentosaceus* Strains					*P. acidilactici* Strains				
	MI120	MI121	MI122	MI123	MI124	MI125	MI126	MI127	MI128	MI129	MI130
*B. cereus* ATCC 11778	100.00 ± 0.00	50.00 ± 0.00	50.00 ± 0.00	25.00 ± 0.00	25.00 ± 0.00	25.00 ± 0.00	50.00 ± 0.00	50.00 ± 0.00	25.00 ± 0.00	25.00 ± 0.00	25.00 ± 0.00
*E. coli* ATCC 8739	100.00 ± 0.00	50.00 ± 0.00	50.00 ± 0.00	25.00 ± 0.00	25.00 ± 0.00	25.00 ± 0.00	50.00 ± 0.00	50.00 ± 0.00	25.00 ± 0.00	25.00 ± 0.00	25.00 ± 0.00
*Kocuria cristianae* MI20	3.13 ± 0.00	3.13 ± 0.00	100 ± 0.00	50.00 ± 0.00	50.00 ± 0.00	50.00 ± 0.00	100.00 ± 0.00	100.00 ± 0.00	50.00 ± 0.00	50.00 ± 0.00	100.00 ± 0.00
*L. monocytogenes* ATCC 13932	12.50 ± 0.00	50.00 ± 0.00	25.00 ± 0.00	50.00 ± 0.00	25.00 ± 0.00	50.00 ± 0.00	50.00 ± 0.00	50.00 ± 0.00	50.00 ± 0.00	50.00 ± 0.00	50.00 ± 0.00
*P. aeruginosa* ATCC 15442	50.00 ± 0.00	12.5 ± 0.00	12.50 ± 0.00	6.25 ± 0.00	6.25 ± 0.00	6.25 ± 0.00	6.25 ± 0.00	6.25 ± 0.00	6.25 ± 0.00	6.25 ± 0.00	12.50 ± 0.00
*R. equi* ATCC 6939	50.00 ± 0.00	50.00 ± 0.00	25.00 ± 0.00	12.50 ± 0.00	25.00 ± 0.00	25.00 ± 0.00	50.00 ± 0.00	25.00 ± 0.00	12.50 ± 0.00	25.00 ± 0.00	25.00 ± 0.00
*P. vulgaris* ATCC 13315	25.00 ± 0.00	6.25 ± 0.00	6.25 ± 0.00	6.25 ± 0.00	25.00 ± 0.00	6.25 ± 0.00	6.25 ± 0.00	3.13 ± 0.00	3.13 ± 0.00	6.25 ± 0.00	6.25 ± 0.00
*S.* Typhimurium ATCC 14028	100.00 ± 0.00	25.00 ± 0.00	50.00 ± 0.00	12.50 ± 0.00	25.00 ± 0.00	12.50 ± 0.00	25.00 ± 0.00	12.50 ± 0.00	25.00 ± 0.00	25.00 ± 0.00	25.00 ± 0.00
*S.* Enteritidis ATCC 13076	50.00 ± 0.00	12.50 ± 0.00	12.50 ± 0.00	12.50 ± 0.00	12.50 ± 0.00	12.50 ± 0.00	12.50 ± 0.00	12.50 ± 0.00	6.25 ± 0.00	12.50 ± 0.00	12.50 ± 0.00
*S. marcescens* ATCC 14756	50.00 ± 0.00	25.00 ± 0.00	25.00 ± 0.00	12.50 ± 0.00	12.50 ± 0.00	12.5 ± 0.00	12.5 ± 0.00	12.5 ± 0.00	12.50 ± 0.00	12.50 ± 0.00	12.50 ± 0.00
*S. aureus* ATCC 33592	50.00 ± 0.00	50.00 ± 0.00	12.50 ± 0.00	12.50 ± 0.00	25.00 ± 0.00	12.50 ± 0.00	12.50 ± 0.00	12.50 ± 0.00	12.50 ± 0.00	25.00 ± 0.00	25.00 ± 0.00
*S. aureus* ATCC 6538	25.00 ± 0.00	12.50 ± 0.00	6.25 ± 0.00	6.25 ± 0.00	12.50 ± 0.00	12.50 ± 0.00	12.50 ± 0.00	6.25 ± 0.00	12.50 ± 0.00	12.50 ± 0.00	12.50 ± 0.00
*S. epidermidis* ATCC 51625	50.00 ± 0.00	12.50 ± 0.00	12.50 ± 0.00	6.25 ± 0.00	6.25 ± 0.00	6.25 ± 0.00	12.5 ± 0.00	6.25 ± 0.00	6.25 ± 0.00	6.25 ± 0.00	6.25 ± 0.00
*S. epidermidis* ATCC 12228	50.00 ± 0.00	6.25 ± 0.00	6.25 ± 0.00	6.25 ± 0.00	6.25 ± 0.00	6.25 ± 0.00	6.25 ± 0.00	6.25 ± 0.00	6.25 ± 0.00	6.25 ± 0.00	3.13 ± 0.00

**Table 8:** Total phenolic and flavonoid content of LAB-derived CFS samples.

LAB Strains	CFS Samples	TPC (μg·mL^−1^)	TFC (μg·mL^−1^)
*E. faecium*	MI120	159.99 ± 1.35 ^ab^	21.18 ± 0.72 ^a^
*P. pentosaceus*	MI121	162.18 ± 2.05 ^a^	21.04 ± 0.44 ^a^
	MI122	158.12 ± 2.01 ^abc^	20.56 ± 0.22 ^a^
	MI123	157.11 ± 1.65 ^ad^	17.57 ± 0.17 ^b^
	MI124	151.95 ± 1.57 ^df^	20.93 ± 0.23 ^a^
	MI125	153.19 ± 1.89 ^cde^	21.69 ± 0.72 ^a^
*P. acidilactici*	MI126	152.73 ± 1.85 ^df^	20.28 ± 0.21 ^a^
	MI127	151.77 ± 2.11 ^ef^	21.52 ± 0.75 ^a^
	MI128	156.79 ± 2.22 ^bde^	21.14 ± 0.69 ^a^
	MI129	155.42 ± 2.02 ^bde^	21.79 ± 0.81 ^a^
	MI130	156.34 ± 1.68 ^bde^	20.87 ± 0.18 ^a^

TPC—total phenolic content; TFC—total flavonoid content; ^a–f^—different superscript letters within the column represent significant differences (*p* < 0.05) for the same analysis.

**Table 9:** Antioxidant activity by DPPH of CFS samples.

LAB Strains	CFS Samples	Antioxidant Activity by DPPH		
		Inhibition Percent (%)	IC_50_ (df)	TE (μg·mL^−1^)
*E. faecium*	MI120	75.19 ± 0.37 ^a^	1/12	21.37 ± 0.10 ^a^
*P. pentosaceus*	MI121	70.99 ± 0.21 ^bc^	1/8	20.23 ± 0.06 ^bc^
	MI122	69.75 ± 0.21 ^bc^	1/7	19.89 ± 0.06 ^cd^
	MI123	70.12 ± 0.21 ^bc^	1/7	19.99 ± 0.06 ^bd^
	MI124	66.05 ± 2.35 ^d^	1/4	18.88 ± 0.46 ^e^
	MI125	70.62 ± 0.43 ^bc^	1/9	20.12 ± 0.12 ^bd^
*P. acidilactici*	MI126	68.64 ± 1.71 ^cd^	1/9	19.58 ± 0.47 ^d^
	MI127	70.25 ± 0.43 ^bc^	1/9	20.02 ± 0.12 ^bd^
	MI128	68.77 ± 0.21 ^cd^	1/9	19.62 ± 0.06 ^cd^
	MI129	70.99 ± 0.27 ^bc^	1/11	20.23 ± 0.07 ^bc^
	MI130	72.10 ± 0.57 ^b^	1/11	20.53 ± 0.15 ^b^

df—dilution factor; TE—Trolox equivalent; IC_50_ antioxidant values of CFS were obtained from dose–response curves using GraphPad Prism. ^a–e^—different superscript letters within the column represent significant differences (*p* < 0.05) for the same analysis.

## Data Availability

The original contributions presented in this study are included in the article. Further inquiries can be directed to the corresponding author.
